# Microneedle arrays integrated with living organisms for smart biomedical applications

**DOI:** 10.7150/thno.66478

**Published:** 2021-10-25

**Authors:** Bo Cai, Yusheng Gong, Zheng Wang, Lin Wang, Wei Chen

**Affiliations:** 1 Department of Clinical Laboratory, Union Hospital, Tongji Medical College, Huazhong University of Science and Technology, Wuhan 430022, China.; 2 Department of Pharmacology, School of Basic Medicine, Tongji Medical College, Huazhong University of Science and Technology, Wuhan 430030, China.; 3 Hubei Key Laboratory for Drug Target Research and Pharmacodynamic Evaluation, Huazhong University of Science and Technology, Wuhan 430030, China.; 4 Department of Gastrointestinal Surgery, Union Hospital, Tongji Medical College, Huazhong University of Science and Technology, Wuhan 430022, China.; 5 Research Center for Tissue Engineering and Regenerative Medicine, Union Hospital, Tongji Medical College, Huazhong University of Science and Technology, Wuhan 430022, China.

**Keywords:** microneedle, living organism, smart integration, vaccination, anti-infection, cell-based therapy, cell and secretion delivery

## Abstract

Various living organisms have proven to influence human health significantly, either in a commensal or pathogenic manner. Harnessing the creatures may remarkably improve human healthcare and cure the intractable illness that is challenged using traditional drugs or surgical approaches. However, issues including limited biocompatibility, poor biosafety, inconvenience for personal handling, and low patient compliance greatly hinder the biomedical and clinical applications of living organisms when adopting them for disease treatment. Microneedle arrays (MNAs), emerging as a promising candidate of biomedical devices with the functional diversity and minimal invasion, have exhibited great potential in the treatment of a broad spectrum of diseases, which is expected to improve organism-based therapies. In this review, we systemically summarize the technologies employed for the integration of MNAs with specific living organisms including diverse viruses, bacteria, mammal cells and so on. Moreover, their applications such as vaccination, anti-infection, tumor therapy and tissue repairing are well illustrated. Challenges faced by current strategies, and the perspectives of integrating more living organisms, adopting smarter materials, and developing more advanced technologies in MNAs for future personalized and point-of-care medicine, are also discussed. It is believed that the combination of living organisms with functional MNAs would hold great promise in the near future due to the advantages of both biological and artificial species.

## Introduction

Numerous living organisms play significant roles in human health, some of which develop commensalism with human beings (*e.g.*, gut microbes that extensively interact with the health of the host 1, 2), while others would cause diseases (*e.g.*, pathogenic parasitosis 3). Due to the development of biological and material science, various organisms have been harnessed and utilized directly or in a modified manner to improve human healthcare. One famous successful case is the invention of live-attenuated vaccines 4. Since the inoculation of cowpox that was popularized by Edward Jenner in the 18^th^ century and the observation of attenuated principles for bacteria by Louis Pasteur, attenuated viruses or bacteria vaccinated to induce acquired immunity and establish immune prevention have saved countless lives 5, 6. Some other living organisms have also been employed to develop novel therapeutic strategies for the diseases that are difficult to cure by traditional treatments. For instance, reconstitution or transplantation of microbes in the gut was able to relieve symptoms of recurrent clostridium difficile infection, Crohn's disease, ulcerative colitis, and so on 7-9. Other successes include cell-based therapies that use autologous/allogeneic adult cells or stem cells (such as mesenchymal stem cells (MSCs) or induced pluripotent stem cells (iPSCs)) or function-engineered cells to repair injured or pathological tissues or organs, some of which have been applied in diseases relating to heart, liver, kidney, skeleton, nerve, eye, and so on 10-12. Therapies using engineered immune cells have achieved remarkable success in treating cancers like leukemia and melanoma 13, 14. Based on the discussion above, living organism-based therapy has become one of the most important subcategories in the field of biological medicine, undoubtedly attracting increasing attention from the entire world.

Compared to traditional therapies based on drugs or surgery, employing living organisms enables long-term disease treatment as a result of continuous interaction between the creatures and human bodies. Some of them persistently produce secretions such as cytokines, triggering protection or repairing, while others target specific cells or lesions for antigen or drug delivery. Furthermore, the abilities of proliferation and differentiation of some organisms (mainly stem cells) exhibited the promising potential to repair or replace cells or tissues that have lost functions when injured or in pathological states 15-17. However, therapies using living organisms encounter several challenges: (1) Viability and safety. On one hand, the colonization of exogenous creatures into the human body confronts the immunological rejection from the host which hinders their survival. On the other hand, the organisms may proliferate out of control, and thus leading to tumorigenesis/parasitosis, or severe immune reaction to threaten lives 18, 19. (2) Inconvenience and low compliance. Currently, therapeutic organisms are mainly delivered *via* bolus injection (*e.g.*, vaccination 20 or cancer therapy 21), scaffold transplantation (*e.g.*, the use of cardiac patches 22), or even surgical methods 23, which presents challenges toward personalized medicine at home or anywhere besides hospitals, and thus compromising patient compliance, greatly impeding their translation to clinical applications. Therefore, a safe and easy-to-use treatment regime that does not require professionals or specialized facilities is highly demanded.

With the development of novel materials and micromachining techniques, the emerging microneedle technology has become a promising candidate to treat various diseases in a minimally invasive manner without sophisticated equipment or specific professionals 24-27. Microneedle array (MNA) is a piece of a substrate (mm^2^ to cm^2^ scale) containing an array of needles (a few to several hundred tips in total) with diverse mechanical and physicochemical properties. The individual needle on MNAs is well-shaped (*e.g.*, cone or pyramid) with the length ranging from 200 to 1500 μm, and the diameter is around 250 to 600 μm 28, 29. By integrating with biosensing systems or drug formulations, MNAs have been widely applied in biomarker detection (*e.g.*, interstitial fluid or blood detection based on chemical, electrochemical or immune strategies) and specific drug delivery (such as delivering small molecules, peptides, proteins, oligonucleotides, nanoparticles, and so on) 30-32. With advantages of minimal invasion, easy operation and bioresponsive delivery strategies, MNAs significantly benefit personal drug administration and improves patient compliance, and therefore demonstrating excellent strategies to meet challenges raised in current therapies using living organisms.

**Figure 1:**
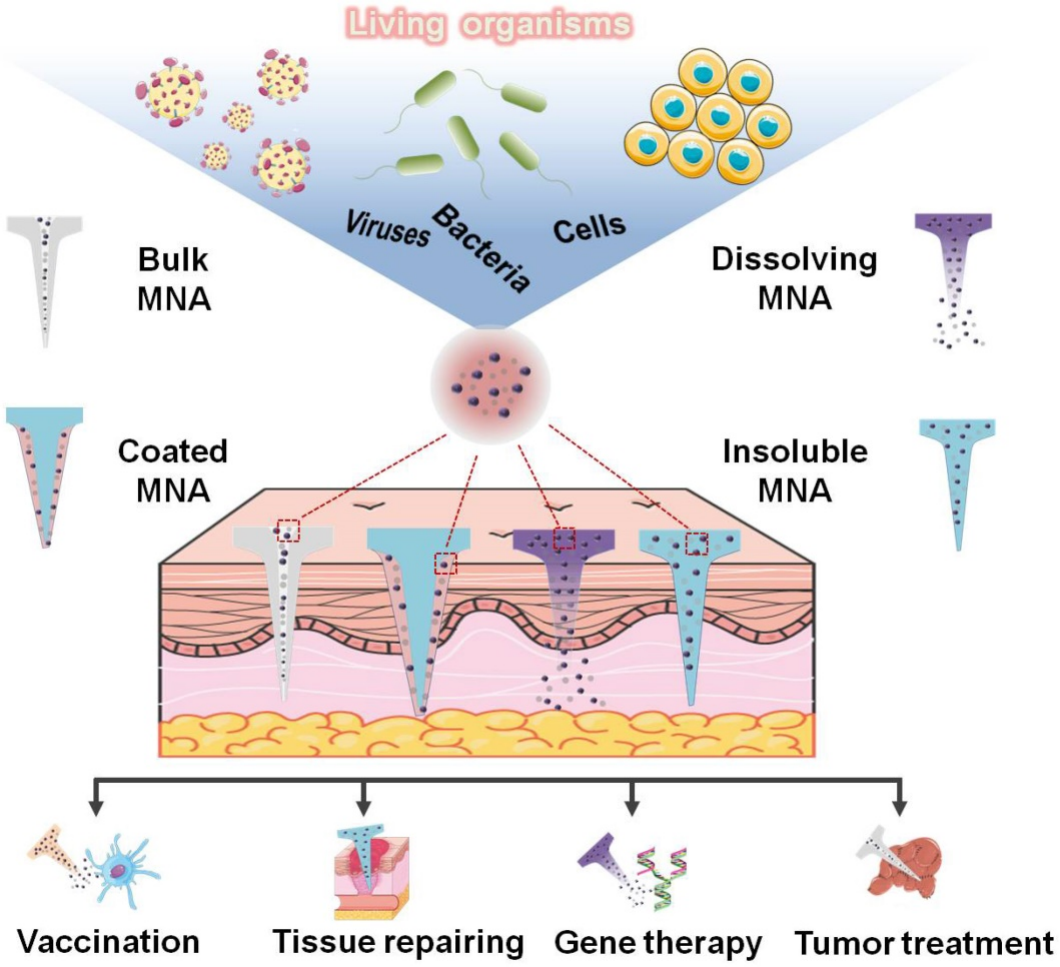
Specific living organisms including diverse viruses, bacteria, and mammal cells are integrated into MNAs and are widely applied in the fields of vaccination, tissue repairing, gene therapy, tumor treatment, and so on.

In this review, we systemically summarize the combinative systems of MNAs and living organisms, with specific biological species including diverse viruses, bacteria, mammal cells, and so on. Moreover, their applications such as vaccination, anti-infection, tumor therapy, and tissue repairing are well illustrated (**Figure 1**). Initially, we briefly introduce the specific classification and materials of MNAs used to integrate organisms. Subsequently, detailed integration strategies using the living organism and MNAs, and their corresponding applications for drug delivery and disease treatment are categorized according to the species of the organisms (*i.e.*, virus, bacteria and mammal cells). Finally, we highlight the challenges faced by current strategies, and the perspectives including more living organisms, smarter materials and advanced novel technologies are discussed in-depth.

## Typical MNAs for living organism integration

Strategies based on organism-MNA combination are mainly divided into two categories: (1) Directly applying living organisms into tissues/organs to induce repairing effects or the immune response. (2) Utilizing the secretion of living organisms to mediate metabolism or eradicate pathological cells/bacteria. Given the different cargoes (living species or their secretion) that needed to be delivered, MNA forms with distinct constitutions and working mechanisms are optimized. Moreover, the material selection for organism-integrated MNA fabrication should include the following features: (1) Suitable stiffness to ensure tissue penetration. (2) Excellent biocompatibility with minimal damage to the hosts and the integrated organisms. (3) Sufficient loading capacity of therapeutic agents for disease treatment.

Currently, there are mainly four types of MNAs, including bulk, coated, dissolving and insoluble MNAs. Each type of these MNAs adopts specific materials for fabrication and has its own unique delivery profile 32, 33, and they can be employed flexibly according to the requirement of integrating different organisms for special applications.

Bulk MNAs are mainly made of stiff materials such as metal (*e.g.*, stainless steel), silicon and stiff polymers (*e.g.*, poly(L-lactic acid)) with or without channels inside the MNAs. They are able to form micro-holes on the surface of target tissues to facilitate the therapeutic agents' transport. Among all types of MNAs, bulk MNAs display the highest mechanical strength, ensuring successful insertion and thus guaranteeing the delivery efficiency. Therefore they are widely employed for virus/bacteria-based vaccination.

Differently, coated and dissolving MNAs would dissolve into target tissues to release the encapsulated organisms after penetration. For coated MNAs, agents such as drugs or regulatory factors are directly deposited on the surface of MNA tips, which enables well mixture of the payload with interstitial fluid (ISF) to improve their diffusion into target locations. Meanwhile, dissolving MNAs are made of dissolvable materials (*e.g.*, saccharides) that encapsulate therapeutic agents inside. After inserting into tissues, the whole microneedles are dissolved in ISF and release the payloads. In order to trigger ignorable immunogenicity or toxicity when degraded, and to effectively maintain the viability of organisms in MNAs, natural extracts like carboxymethyl cellulose (CMC), hyaluronic acid (HA) and saccharides (*e.g.*, dextran, sucrose, maltose), or synthesized polymers such as polyvinyl alcohol (PVA), polyvinyl pyrrolidone (PVP) and poly(ethylene glycol) (PEG) with benign biocompatibility are commonly employed 34. After proper chemical modification, these materials can be stimuli-responsive (pH, temperature, light and so on) and therefore endow MNAs with smart functions (*e.g.*, spontaneously responding to blood glucose variation) 35, 36. Compared to bulk MNAs, these MNAs present superior advantages of simple manufacture, low cost, and excellent biocompatibility, demonstrating the powerful capability of controllable drug delivery/release and stability/viability maintenance of living organisms.

In cases that living organisms need to retain inside MNAs to continuously secrete therapeutic agents and escape from the host immune response, insoluble MNAs encapsulating the creatures would be a preferred candidate owning to their biostability and porous structures. These MNAs are mainly made of insoluble hydrogels that can enclose therapeutic agents inside. Due to the porous and cage-like structure of the hydrogel, large payloads are retained inside the hydrogel while small molecules are continuously released outside, which can specifically regulate the secretion behavior of living species. For this purpose, the aforementioned biocompatible hydrogel is usually modified and crosslinked to be the insoluble form, providing long-term treatment. After penetrating target tissues, the MNAs spontaneously swell rather than dissolve, and thus maintain the organisms inside the MNAs while allow secretion to diffuse through the interspace of the hydrogel molecules.

## MNAs integrated with living organisms

### MNAs integrated with virus

Despite the tiny size (at the nanometer scale) and simple structures (only including nucleic acids and proteins), the viruses have been recognized as highly dangerous organisms given their high pathogenicity and infectivity 37, 38. Numerous diseases relate to specific viruses, and some of them are still intractable, such as human immunodeficiency virus (HIV), Zika, coronavirus disease 2019 (COVID-19), and so on. Since Edward Jenner demonstrated the prevention of smallpox by prior infection with less dangerous cowpox virus in 1796 39, the vaccine that utilizes exterior proteins/subunits of viruses to induce the virus recognition/clearance by the innate immune system, has been widely used to prevent virus-induced illness 40. The conventional vaccine is mainly implemented *via* the hypodermic needle injection, which faces challenges including pain, cold-chain storage, hazardous sharps-waste, and the tremendous demand of trained medical personnel 41, 42. MNAs, a promising alternative to needle injection, are painless, inexpensive and easy-for-use, which can act as an ideal tool for vaccine inoculation. Moreover, after adopting proper formulation, the virus vaccine loaded in MNAs can maintain long-term stability even in room temperature 43. Besides vaccines, viruses have proven to work as vectors for gene delivery or direct tumor treatment 44-46. After integration with MNA technology, the delivery of these kinds of viruses would be greatly improved, therefore facilitating gene therapies and tumor treatments. Applications using MNAs integrated with viruses are summarized in **Table 1**.

Recombinant viruses, which are alive but non-replicating, can be gene-edited by inserting exogenous genes that encode antigens of malaria, influenza, tuberculosis, HIV, and so on, serving as effective vaccines 64, 65. For example, modified vaccinia virus Ankara (MVA) and replication-defective adenovirus (*e.g.*, human adenovirus serotype 5 (AdHu5) and chimpanzee serotype 63 (ChAd63)) have been confirmed to be safe and immunogenic in human clinical trials 66, 67. Vaccines from these living recombinant viral vectors have demonstrated great promise based on their capability to induce strong T-cell immunity 68, 69.

**Figure 2:**
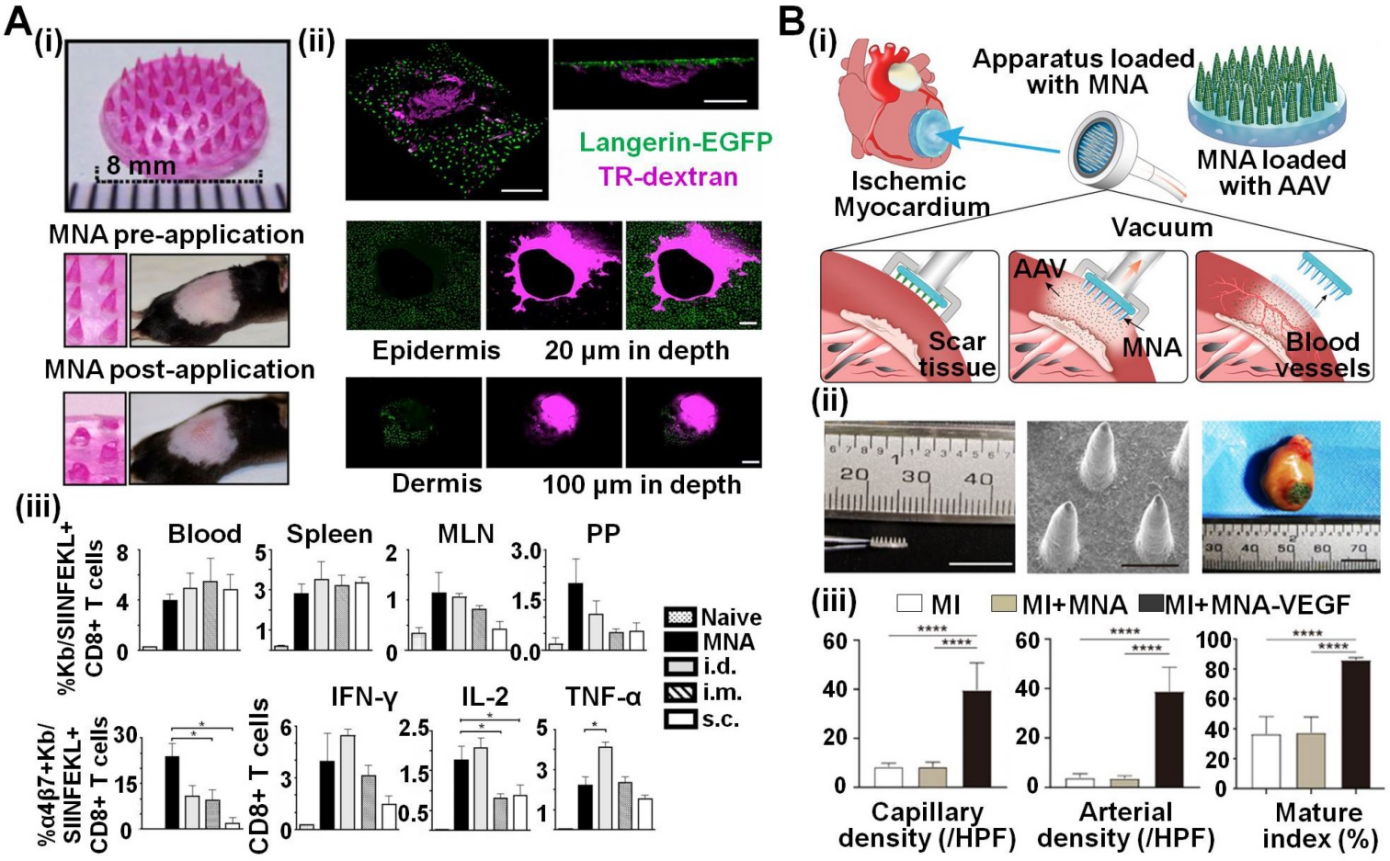
** Recombinant viruses integrated into MNAs. A (i)** A dissolving MNA preserving the immunogenicity of dried AdHu5.** (ii)** Representative intravital confocal images of epidermis and dermis after MNA application, showing excellent MNA penetration into the skin with rapid dissolution.** (iii)** The MNA application-induced immunization response equipotent with conventional injected routes of vaccine delivery. Reproduced with permissions from National Academy of Society, USA 2013 49. **B (i** and** ii)** Ischemic hearts were treated by an MNA-encapsulating recombinant AAV9 which enabled gene transfection for enhancing VEGF expression, promoting functional angiogenesis and activating the Akt signaling pathway. **(iii)** MNAs delivered AAV-VEGF into the tissues of the infarction and enabled the local neovascularization. Reproduced with permissions from American Association for the Advancement of Science 2020 55.

MNA technology has been widely applied for recombinant virus delivery to induce an intense immune response, and bulk MNAs are usually utilized given their excellent delivery efficiency. Moore *et al*. developed silicon MNAs with different dimension-related parameters, and compared their influence on the magnitude and memory of vaccine-induced CD8+ T cells 47. Pearson *et al*. from the same research group also delivered an adenovirus-vectored malaria vaccine using the identical bulk MNAs, achieving corresponding results 52. In the regime of bulk MNA delivery, vaccines were placed on the skin, followed by MNA treatment, which may lead to inconvenient operation and inaccurate dosage control. Therefore, coated MNAs became a more popular choice due to their low cost, one-step application and precise dose control. Intriguingly, Moore *et al*. investigated a spray-coating technique to load vaccines onto MNAs 48. Different saccharides formulations were included to maintain the viral activity, acting as effective adjuvants. A similar investigation was implemented on MNA modified by dry-coating technology, and the data suggested the long-term thermostability of malaria vaccines loaded in MNAs over traditional ones 51. Besides, dissolving MNAs were also employed to deliver recombinant viral vaccines due to their quick release and functional diversity. Klavinskis *et al*. adopted sodium carboxymethyl cellulose (Na-CMC) to fabricate a dissolving MNA containing AdHu5-vectored vaccine, which effectively exerted functions for at least one month. Their work also revealed the critical roles of different immune cells performed during the MNA vaccination (**Figure 2A**) 49. A similar immune response was observed when applying dissolving MNAs to deliver vaccines against malaria and HIV 50, 53, 54.

MNAs are also utilized to deliver recombinant viruses as gene carriers toward treating diseases based on gene therapy. Ye *et al*. engineered phase-transition MNAs loaded with adeno-associated virus (AAV) to deliver genes of vascular endothelial growth factor (VEGF) for cardiovascular disorder treatment (**Figure 2B**) 55. The delivered AAV transfected cardiomyocytes in a homogeneous manner and was distributed evenly in the myocardiums. These MNAs significantly enhanced VEGF expression, promoted functional angiogenesis and thus improved heart functions in the ischemic area.

**Figure 3:**
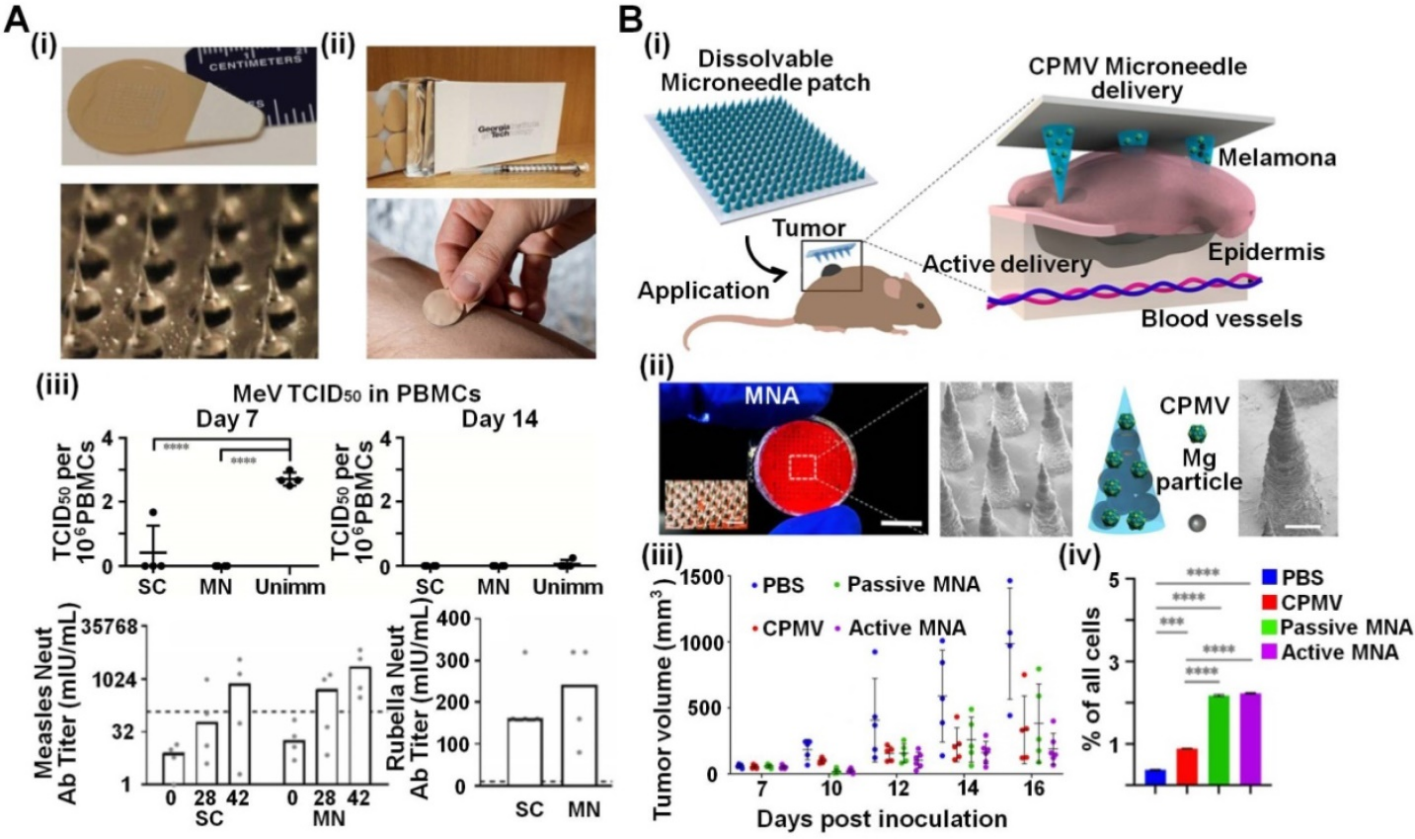
** Live viruses integrated into MNAs used for vaccination or tumor treatment. A (i)** Dissolving CMC MNAs containing live-attenuated Edmonston-Zagreb and RA-27 virus strains as the vaccine for measles and rubella. The MNAs made a minimal invasion on the skin **(ii)** while induced effective vaccination equipotent with subcutaneous injection and protected infant rhesus macaques from challenges with wild-type viruses **(iii)**. Reproduced with permissions from Elsevier Limited 2020 59. **B (i)** *In situ* vaccination using an autonomous MNA for the treatment of B16F10 melanoma by releasing plant virus nanoparticles. **(ii)** The MNAs were fabricated using water-soluble polymer of PVP and contained Mg microparticles for active delivery of therapeutic CPMV nanoparticles. **(iii)** Tumor growth of melanoma on mice could be effectively suppressed, mainly by **(iv)** CPMV-induced *in situ* vaccination via enhancement of intratumor recruitment and activation of live CD45^+^ cells. Reproduced with permissions from American Chemical Society 2020 63.

Besides recombinant viruses, natural viruses themselves have been broadly employed for infectious disease prevention and tumor treatment. Because replication competence and viral virulence do not frequently correlate with immunological robustness, live-attenuated viruses could also serve as potential vaccines. After attenuation, virus toxicity would decrease but their immunogenicity is reserved so that they can still trigger the immune response effectively 65. Due to convenient operation and thermostability, attenuated virus-loaded MNAs remarkably improve vaccine storage and efficacy, especially in developing countries. Prausnitz *et al*. engineered MNAs to administer measles and rubella (MR) vaccine in rhesus macaques models (**Figure 3A**) 57-59. With the help of sucrose-based MNAs, MR vaccines can be administered without well-trained personnel, resulting in fewer biohazardous sharp wastes and absent cold-chain storage. Similarly, various live-attenuated viruses including mumps, vaccinia and dengue have been successfully loaded in specific MNAs, and have proven to work as efficient vaccines 56, 60-62.

In addition to vaccines, viruses could also be applied to treat tumors (*e.g.*, oncolytic viruses) 44. Considering that MNAs can easily penetrate the skin, a combination of virus and MNAs to treat melanoma was implemented by Wang *et al*. (**Figure 3B**) 63. They directly delivered cowpea mosaic virus (CPMV) intratumorally by MNA assisted by Mg nanoparticles, significantly suppressing canine melanoma in mice models. Notably, after delivery by dissolving MNAs, CPMV could intensively activate the innate immune system and recruit effecter T cells to suppress tumors. This work obviously confirmed the possibility of immune-mediated antitumor effects with the help of transdermal virus-integrated MNAs that can be easily implemented without professionals.

### MNAs integrated with bacteria

It is well known that bacteria have shown a complicated relationship with human health for years 70. Some so-called “probiotics” develop commensalism with human beings, and stay in organs such as the skin and gastrointestinal tract for a long time. Interestingly, they help human maintain homeostasis, digest food, provide unique metabolites and even benefit immunity. Meanwhile, there are still some bacteria causing various diseases especially when they are out of control 71-74. However, those “harmful” bacteria can also be utilized to “train” our immune system to obtain specific immunity, suggesting potential infection prevention 75, 76. Harnessing bacteria will undoubtedly benefit disease prevention and treatment with the help of artificial intervene. As the fast-developed approach for “smart” delivery of therapeutics, MNAs provide a facile and convenient tool to improve bacteria-based formulation in biomedical applications (**Table 2**).

**Figure 4:**
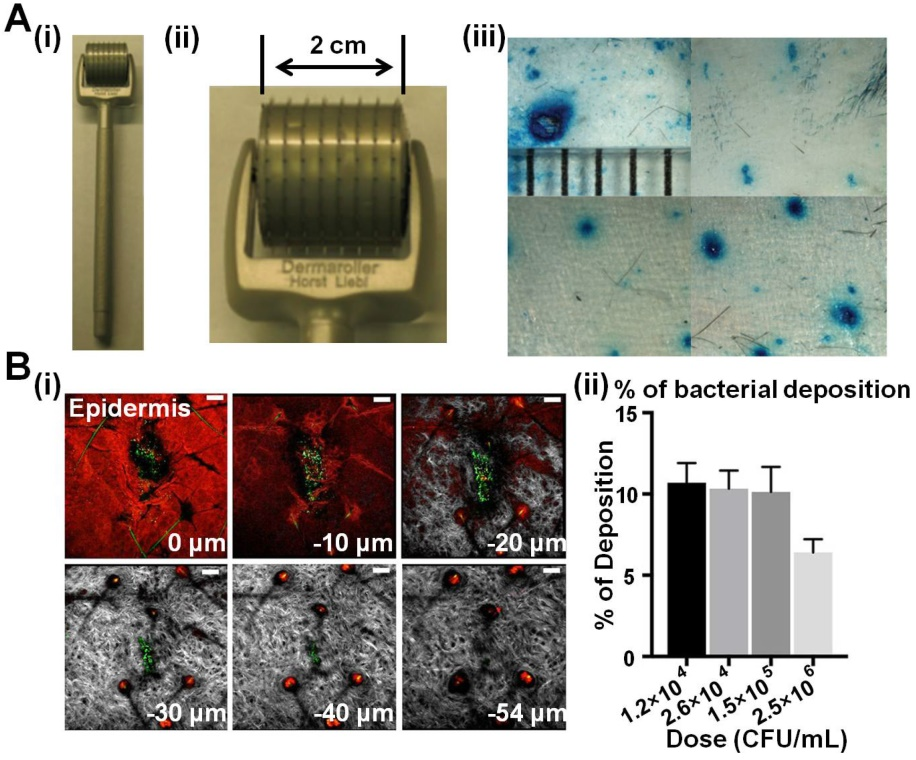
** Bulk MNAs for intradermal bacteria permeation. A** A commercial Dermaroller^®^ MNA roller **(i** and **ii)** was used to evaluate *E. coli* DH5α penetration through MNA-treated mouse skin. **(iii)** Lesions made by a hypodermic needle and MNAs with needles of small (200 µm), medium (500 µm) and large (1000 µm) sizes were compared. Reproduced with permission from Dove Medical Press Limited 2011 78. **B (i)** Green fluorescent microspheres were employed to simulate *F. novicida* delivery to the ear skin of a DsRed-transgenic mouse using bulk PLLA MNAs, and** (ii)** the percentage of viable bacteria deposited from the MNA could be enumerated. Reproduced with permission from American Society for Microbiology 2018 79.

Living bacteria can be applied to evaluate the damage of the skin triggered by bulk MNAs. Donnelly *et al*. employed a silicon MNA on an *in vitro* model (excised porcine skin) to assess the infection potential of lesions compared with hypodermic needles 77. They coated microbes (*i.e.*, *C. albicans*, *P. aeruginosa* and *S. epidermidis*) on the surface of skin models and punctured the skin with hypodermic needles or MNAs. Subsequently, the number of bacteria in the skin was calculated to evaluate the possibility that organisms traversed the MNA-induced channels. Results obviously demonstrated that MNAs would not result in severe local or systemic infections, and thus validating the minimal invasion and safety of MNAs. Cui *et al*. obtained similar conclusions using a commercial MNA roller (Dermaroller^®^) made of metal (**Figure 4A**) 78. In order to gain more information on how the delivered bacteria trigger the host response, Ernst *et al*. tested them in mice models (**Figure 4B**) 79. *F. novicida*, a kind of bacteria that caused tularemia, was adhered onto a bulk MNA made of poly(L-lactide acid) (PLLA), and applied onto C57BL/6 mice. Successful bacteria deposition was observed, and survival rate and tissue response (in spleen, liver, lung and blood) such as T-cell depletion and inflammatory cytokine levels were monitored. Their results evidently showed that MNAs could effectively deliver bacteria into the skin to regulate immune responses *in vivo*.

With advances of biomaterials that could endow MNAs with smart functions like stimuli-responsiveness and sustained release, MNAs could be applied to significantly enhance bacteria-based therapies 29, 30. For instance, aiming to popularize the inoculation of tuberculosis vaccine in areas with low economy and slow development, Wu *et al*. invented a dissolving MNA that contained caves near the bases of needles to store live-attenuated Bacille Calmette-Guerin (BCG) *bacillus* as a vaccine 80. Tests showed that BCG *bacillus* could be stored for at least 90 days at room temperature, and was able to induce desirable immune protection, validated by intracellular cytokine productions and humoral responses in mice models. This MNA-based strategy exhibited similar inoculation efficiency compared to traditional intramuscular injection, while it did not require any medical professionals/equipment and cold-chain transportation, therefore facilitating popularization.

**Figure 5:**
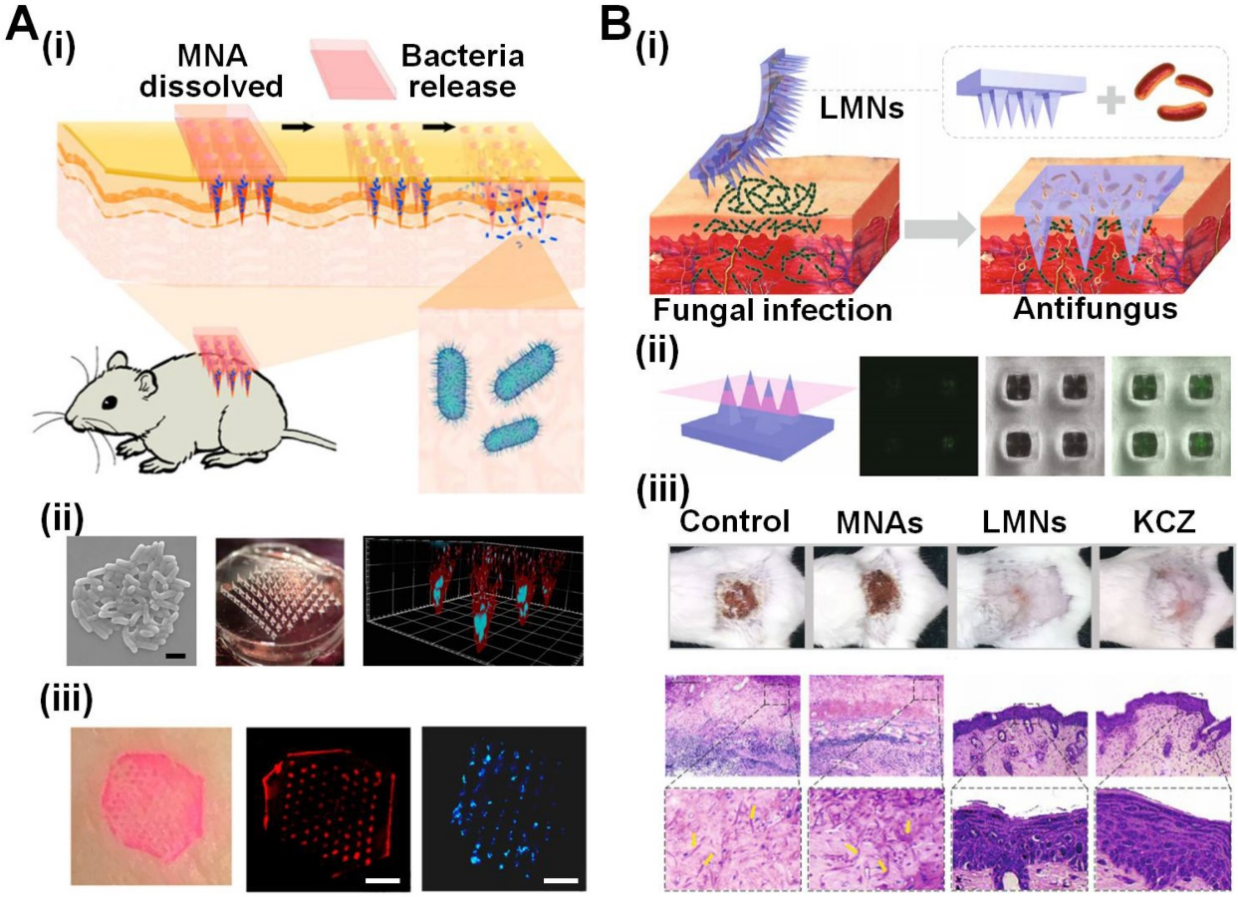
** MNAs encapsulating therapeutic bacteria in a dissolving or hydrogel-forming manner. A (i)** MNAs delivering bioactive functional probiotics into local skin to improve skin health and immunity. **(ii)** *Lactobacillus* as the model probiotic were encapsulated in the MNA tips. **(iii)** After insertion, the dissolved sodium carboxymethyl cellulose (SCMC, red fluorescence) and *Lactobacillus* (blue fluorescence) were deposited into the pig skin. Reproduced with permission from American Chemical Society 2018 81. **B (i)** A hydrogel-forming MNA encapsulating *B. subtilis* to release lipopeptides for treating fungal infection. **(ii)** The living bacteria stayed inside the MNAs without invasion and could be removed together with the patch. **(iii)** These MNAs showed effective capability when treating fungi-infected skin similar to ketoconazole. Reproduced with permission from American Association for the Advancement of Science 2020 82.

Other than being used as vaccines, bacteria, especially those probiotics, are also integrated into MNAs to treat skin diseases (*e.g.*, fungal infection). Xie *et al*. encapsulated *lactobacillus* in the dissolvable sodium carboxymethyl cellulose and subsequently micromolded MNAs, followed by local delivery into the skin (**Figure 5A**). Results clearly showed that the probiotics inside the MNAs were kept alive at 4 ℃ for at least 21 days, and they could continuously produce lactic acid for 2 hours, and would not cause subcutaneous inflammation.

Given that some bacterial secretion may act as new agents to treat diseases 70, 71, bacteria are encapsulated in insoluble MNAs to continuously deliver bacterial secretion as therapeutic agents. These MNAs can trap the bacteria inside, and therefore avoid biosafety issues of uncontrolled bacterial proliferation when directly injecting bacteria into the human body. For example, considering the competition between beneficial bacteria and other microbes, Zhao *et al*. proposed a hydrogel-forming MNA that encapsulates living *B. subtilis* to secrete anti-fungal lipopeptides against *C. albicans* infection (**Figure 5B**) 82. The beneficial bacteria were long-term maintained inside the MNAs and therefore would not cause an undesirable immune response, indicating a safe treatment. Importantly, this bacteria-MNA combinative strategy displayed similar antifungal effects compared to antibiotic ketoconazole, while showing no risk of drug resistance.

### MNAs integrated with mammal cells

Recently, cell-based therapies emerge as a fast-growing field, and present great potential in tissue/organ repairing/regeneration and cancer treatment 14, 15. However, traditional cell-based therapies are frequently compromised by low cell viability and immune rejection between hosts and the cells 83, 84. How to achieve precise and controllable delivery of target cells in a convenient manner remains challenging.

Typical MNAs that could be employed for cell delivery are summarized in **Table 3**. Assisted by abundant functional materials, MNAs are able to encapsulate versatile cells and deliver them to various tissues, and therefore benefit the advance of point-of-care cell therapies. Based on the locations of the cells in the MNAs, these systems can be divided into cell-delivery MNAs and secretion-delivery MNAs.

Bulk MNAs have been attempted to deliver mammal cells by creating microchannels on the surfaces of target tissues. Huang *et al*. tried to deliver skin cells (keratinocytes HaCaT and human follicle dermal papilla cells) using bulk polymethyl methacrylate (PMMA) MNAs, which were successfully applied onto *in vitro* model (collagen hydrogel) 85. Cells in suspension were laid and attached on the top of an MNA for 1-4 h and subsequently were transplanted to 2% collagen hydrogel by applying the MNA. The cell transplantation efficiency was around 80%, confirming the feasibility of cell delivery by bulk MNAs. And the cell viability could maintain at about 80% at Day 3 after delivery. Furthermore, cells could be delivered to the target through the channels inside the MNAs made of SiO_2_ or Si_3_N_4_ 86, 87. Cellular viability and functions were evaluated to be similar to those injected by conventional hypodermic needles.

**Figure 6:**
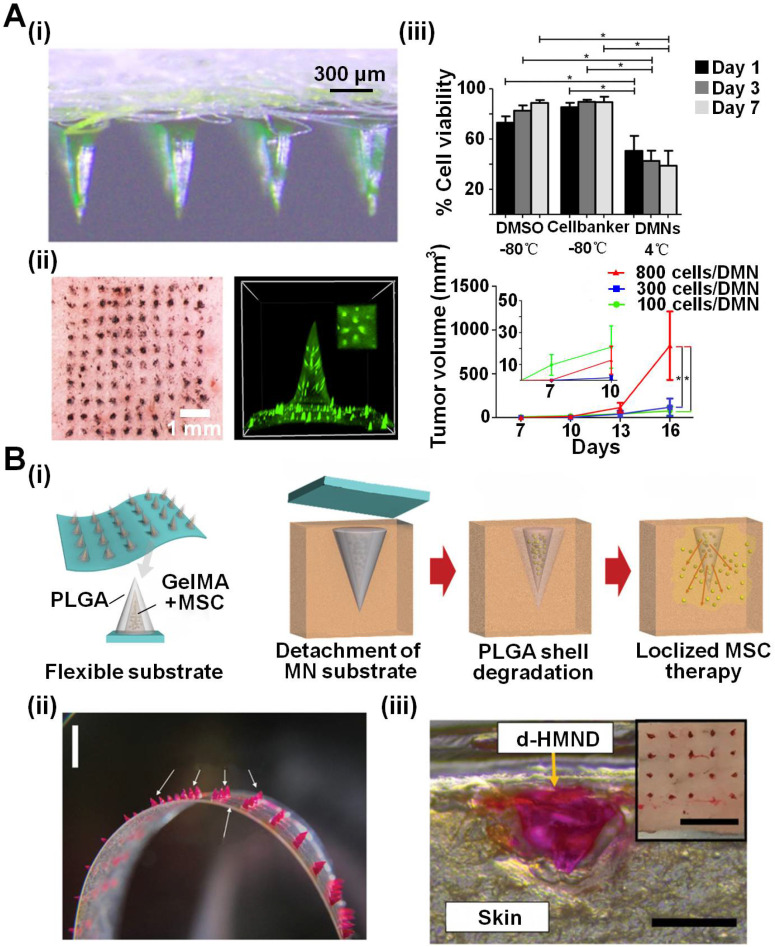
** Transdermal delivery of living therapeutic cells using MNAs for cell-based therapies. A (i** and **ii)** A dissolving MNA made of hyaluronate (HA)/polyvinyl pyrrolidone (PVP)/maltose delivering cancer cells into mice models for tumor grafting. **(iii)** The MNAs could maintain the viability of the encapsulated B16-F10-murine-melanoma at about 38.8% for 7 days. Cell transplantation using these B16-F10-cell-loaded MNAs displayed higher tumorigenesis efficiency and larger tumor formation using less cells compared to the hypodermic needle injection. Reproduced with permissions from American Chemical Society 2020 88. **B (i)** MSCs were delivered into localized skin by a core-shell MNA for would healing. **(ii** and** iii)** Cells were encapsulated in the GelMA hydrogel and coated by PLGA. When applied to mouse skin, PLGA dissolved and MSCs were left inside the target tissue. Cell viability could maintain 24 hours inside the MNA. Reproduced with permission from WILEY-VCH Verlag GmbH & Co. KgaA 2020 89.

Compared to bulk MNAs, the utilization of biocompatible materials may provide high tunability to mediate cell behaviors. In these kinds of MNAs, cells were frequently encapsulated in needle tips and then were directly released in the tissues after penetration. For example, Wanichwecharungruang *et al*. utilized dissolving MNAs made of hyaluronate (HA)/polyvinyl pyrrolidone (PVP)/maltose to deliver cancer cells into mice models for tumor grafting (**Figure 6A**) 88. After drying at 4 °C, the MNAs could maintain the viability of the encapsulated B16-F10-murine-melanoma at about 38.8% for 7 days. Cell transplantation using these B16-F10-cell-loaded MNAs displayed higher tumorigenesis efficiency and larger tumor graft compared to the hypodermic needle injection, possibly due to the temporary protection of HA/PVP/maltose on cells against host immune response. Khademhosseini *et al*. achieved localized MSC therapy using a similar cell-based dissolving MNA strategy (**Figure 6B**) 89. Double-layered MNAs with needle tips that were comprised of a core made by gelatin-methacryloyl (GelMA) containing mesenchymal stem cells (MSCs) and poly(lactic-co-glycolic acid) (PLGA) shell, were employed to improve skin wound healing. After optimizing the fabrication process and constitutes, the MNAs presented superior mechanical property, and maintained the MSC viability over 90% for at least 24 h at 36.5 °C. Importantly, the encapsulated MSCs kept their stemness, and they could secrete vascular endothelial growth factor (VEGF) normally. When applied to the skin wounds, those MNAs showed enhanced healing ability with smaller wound areas, higher re-epithelialization, longer migrating epidermal tongue (MET), and greater CD31 intensity.

Another interesting transdermal cell delivery *via* MNAs was developed by Xu *et al*. using a cryogenic method 90. The MNAs were fabricated by stepwise micromolding of cryogenic medium with pre-suspended cells, which could be readily inserted into porcine skin and dissolved after deployment of the cells. *In vivo* models validated that cells delivered by those cryomicroneedles remarkably retained their viability (ranging from 21.4% to 53.1%) and proliferative capability. In mice models with subcutaneous melanoma tumors, the delivery of ovalbumin-pulsed dendritic cells *via* the cryomicroneedles elicited higher antigen-specific immune responses and led to slower tumor growth compared to intravenous and subcutaneous injections of the cells. These biocompatible cryomicroneedles exhibited great potential to facilitate minimally invasive cell delivery and improve patients' compliance for a range of cell therapies (*e.g.*, CAR T cell therapy 94).

Other typical cell-based MNAs deliver not cells but their secretion (*e.g.*, cytokines) into the pathological tissues to treat corresponding diseases. In those MNAs, functional cells are frequently encapsulated inside the base parts or on their surfaces, and thus are protected by cross-linked insoluble hydrogels against the host immune system. The needle tips of the MNAs create “microchannels” on the surfaces of target tissues and then the secreted therapeutic agents diffused through the microchannels to the pathological locations. Due to the continuously viability maintenance of the encapsulated cells, the cellular secretion persistently treats the lesions for a long time.

**Figure 7:**
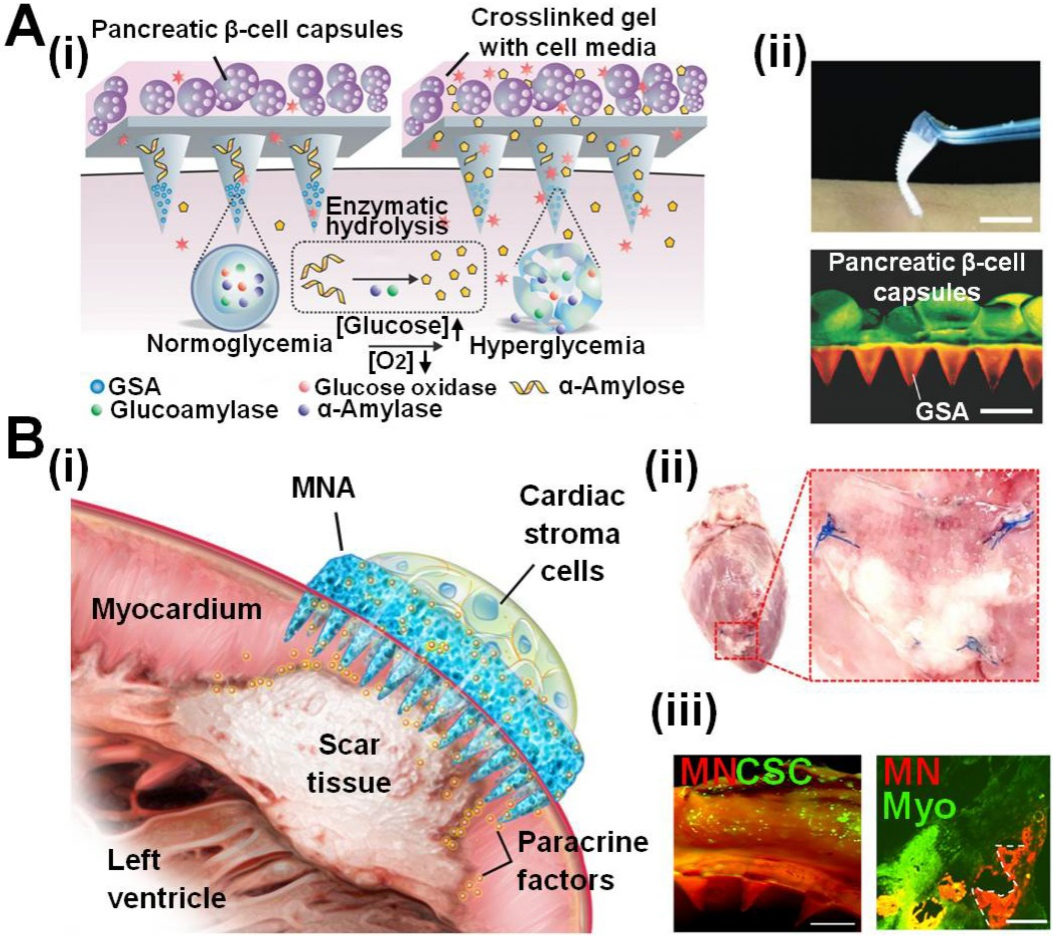
** MNAs encapsulating functional cells for releasing therapeutic agents to treat diseases. A (i)** A Pancreatic β-cells integrated hydrogel-forming MNA to release insulin for treating diabetes in a smart responsive manner. Functional nanoparticles as glucose signal amplifiers sensed the blood glucose that diffused into the MNA and subsequently generated concentrated “amplified” glucose to trigger pancreatic β-cells for releasing insulin. **(ii)** The cell-based MNA penetration made less invasion in the skin while effectively regulate the blood glucose level in diabetes mouse models. Reproduced with permission from WILEY-VCH Verlag GmbH & Co. KgaA 2016 91. **B (i** and** ii)** A cardiac stromal cell integrated MNA treating myocardial infarction. **(ii)** Regenerative factors secreted by therapeutic CSCs seeded in the microneedle base could be transported to the injured myocardium through microneedle tips, enabling good cell retention and effective integration with host myocardium. Reproduced with permission from American Association for the Advancement of Science 2018 92.

Considering the limitations of direct cell implantation for diabetes treatment such as host immunological rejection, Gu *et al*. encapsulated pancreatic β-cells in the MNA base made of HA/alginate hydrogel to achieve smart glucose response and insulin release, significantly improving Type-1 diabetes therapy (**Figure 7A**) 91. They synthesized a kind of nanoparticles termed “glucose-signal amplifier” (GSA) which could sensitively respond to a hyperglycemic state and trigger the insulin secretion of pancreatic β-cells. This innovative GSA was featured with self-assembled polymeric nanosized vesicles entrapping three enzymes (glucose oxidase, α-amylase and glucoamylase), which overcome the limited glucose diffusion and finally amplified glucose signals, sensitizing pancreatic β-cell response. The recombination of cell therapy and smart-responsive materials helped those MNAs effectively regulate the blood glucose level in the streptozotocin (STZ)-induced type-1 mice.

MNAs can provide ingenious strategies to improve cell-based therapies by preventing immune response and extending treatment periods, for example, in the field of curing heart diseases. Heart-derived cardiac stem/stromal cells can secrete regeneration factors to promote endogenous repair of the injured myocardium. However, the low cell viability by injection and the poor fusion with the host myocardium posed unique challenges for their applications. Cheng *et al*. developed polymeric MNAs integrated with cardiac stromal cells (CSCs) for therapeutic heart regeneration (**Figure 7B**) 92. The MNAs provided multiple channels to allow communications between MNAs and the host myocardium. In this way, CSCs in the MNAs could obtain nutrients from the local environment while releasing paracrine factors to repair the heart. Those MNAs had proven to improve myocardial infarction (MI) treatment in experimental animals (rat and porcine), mainly by promoting angiomyogenesis, reducing scar size, and augmenting cardiac functions for 3 weeks. Other similar MNAs for MI treatment were developed by Zhao *et al*. which consisted of three layers: a drug-loaded MNA (bottom layer), parallel-aligned carbon nanotube (CNT) conductive (middle layer), and a methacrylated gelatin (GelMA) hydrogel scaffold (upper layer) 93. iPSC-derived cardiomyocytes were laid and cultured on the surface of GelMA scaffolds, and could be kept viable and beating for at least 18 days. The secretion from integrated cells together with loaded cytokine drugs effectively promoted the regeneration and function recovery of MI hearts in mice.

## Challenges and perspectives

Given the advantages of minimal invasion and easy operation, MNA technology has shown great potential in the fields of point-of-care drug administration, and convenient personalized diagnosis and therapies. Besides sampling biomarkers and delivering therapeutic agents, integrating with living organisms endows MNAs with versatile abilities from vaccination to cell-based therapies. Viruses, bacteria and mammal cells, whose size finely matches with micro-scaled MNAs, are ideal organisms employed in MNAs, showing high promise for either organism or secretion delivery. Those MNAs have been applied in various fields including but not limited to triggering adaptive immunity, treating infections or tumors, and repairing tissues, which provides convenient alternatives without professionals/equipment compared to traditional therapies based on drugs or surgeries.

### Challenges

Although it has exhibited great potential, the integration of living organisms in MNAs still faces several challenges that limit their development:

(1) Maintaining bioactivity or viability of the integrated living organisms. Despite that various adjuvants (*e.g.*, saccharides) have been adopted for long-term storage of virus-integrated MNAs, how to maintain the viability of more complicated organisms such as bacteria or cells in MNAs remains challenging. Recently, specific hydrogels or cryogenic reagents are under investigation, but they frequently require specific storage conditions of liquid nitrogen or humid incubators, which conflicts with the purposes of using MNAs as ordinary and convenient therapeutic tools in daily life.

(2) The biosafety of inducing living organisms into human beings. Since that MNAs either insert or directly implant into tissues, the contact between organisms and the human body lasts a period of time, either in a direct manner or separated by hydrogels. Therefore, the immunogenicity and the biosafety of those encapsulated organisms have to be considered. Certain organisms possess the ability of fast proliferation (*e.g.*, the embedded bacteria or stem cells), and the infection or tumorigenesis will happen if their proliferation is out of control, posing risks to the host's health. Possible mutations of those evolving organisms during their fast growth, under a pathological environment or caused by implanted materials, might influence the organism's behaviors such as proliferation, differentiation, and so on 95, 96. The behavior of originally encapsulated organisms is frequently under control, while the mutated organisms may proliferate immoderately (*e.g.*, tumorigenesis due to gene mutation for cells). And the fast volume increase of these organisms as individual or group finally break the limitation of MNA structures, leading to their escape into the body and causing physical damages of the located tissues, excessive immune/inflammatory response or even death.

(3) Limited species of organisms and few locations for MNA application. Currently, only limited viruses, bacteria and cells are integrated with MNAs due to a lack of effective strategies and materials to hold organisms or regulate their behaviors. Moreover, current applications of MNAs are mainly restricted to the skin and heart tissues. Lack of feasible and practicable application or adhesion manners is the main reason that hinders the development of MNAs on other tissues.

### Perspectives

In order to face the aforementioned challenges, attempts that devoted into following aspects are expected to further benefit human healthcare in a convenient, low-cost and point-of-care manner:

#### Integrating new species

Considering the versatile functions of extensive organisms and abundant biological tools developed to modify them, new species, besides the aforementioned viruses, bacteria and mammal cells, need to be tested in MNAs to seek novel strategies toward improving healthcare and treating diseases. For example, oxygen-producing algae can be utilized to remiss ischemic diseases that happened in the brain/heart 97 or to combat hypoxic tumors 98, 99. In addition, phages that “eat” bacteria can help treat topical bacterial infections 100, 101. Some other engineered bacteria can also be employed to treat tumors or gastrointestinal diseases 102-104. Integrating MNAs with those organisms presents the potential for implementing fast and convenient organism-based therapies just at home and without the assistance of professionals.

Moreover, certain living creatures that possess immunogenicity could be applied to “train” the innate immune system to prevent various diseases. For instance, studies demonstrate that malaria infection can counteract tumor immunosuppressive microenvironment and inhibit the angiogenesis, growth and metastasis of tumors, which has been preliminarily validated by experiments on mice and human beings 105, 106. It indicates the potential of delivering plasmodium by MNA as a non-invasive manner to combat certain cancers. Moreover, eggs of some parasites which are less virulent pathogenic are also able to trigger “vaccine-like” effects and reduce the risk of subsequent exposure to more pathogenic organisms 107, 108. When being combined with delicate MNA strategies, the behavior and functions of those organisms can be well-regulated to facilitate medical applications.

In addition to the living species mentioned above, more organisms related to nutrition delivery and waste clearance could act as new candidates in MNA-biosystem combinations. For example, engineered bacteria/microalgae are expected to intake wastes such as CO_2_ or urea, and synthesize saccharides to supply nutrition to the target tissue. Furthermore, with the development of tissue engineering, many other human cells (*e.g.*, kidney or liver cells) are anticipated to maintain viability inside MNAs, and then implanted to target tissues for metabolic waste clearance in a well regulatory profile.

#### Adopting new materials

Material science plays a significant role in MNA fabrication. Compared to traditional stiff materials with relatively sole functions (*e.g.*, metal and silicon), abundant functional biomaterials, especially various natural or artificial polymers, have proven to significantly improve the performance of MNAs. Biocompatible materials have demonstrated the success of integrating living organisms with satisfactory viability inside MNAs by providing suitable microenvironments, for example, the adjuvant of saccharides is usually adopted to keep the virus stability when loading them into MNAs for vaccine inoculation. Biocompatible hydrogel (*i.e.*, hyaluronate, polyvinyl alcohol) is mainly employed to encapsulate bacteria. During mammal cell encapsulation, the largest challenge is to maintain their viability in MNAs given the vulnerability of cells *in vitro*. One potential solution is to adopt special materials that are similar to the extracellular matrix for MNA fabrication, such as gelatin-methacryloyl (GelMA). Other methods focus on modifying reagents that are prepared for live cell storage at the low temperature, for example, the commonly-used cryogenic medium of dimethyl sulfoxide (DMSO) is incorporated with sucrose to balance between maintaining cell viability and realizing feasible MNA fabrication. What is more, new functionalized materials have drawn increasing attention in this field: (1) Besides biocompatibility, the newly-developed materials are expected to offer continuous nutrition supply, facilitating long-term creatures storage under room temperature or in a refrigerator. For example, extracts from silk (sericin and fibroin) are demonstrated to have a variety of biological activities for promoting cellular proliferation and inhibiting apoptosis, and thus benefit tissue regeneration 109-111. Researchers have started to fabricate MNAs using this kind of material for wound recovery, drug delivery, vaccination and so on 112-114. (2) The materials should not compromise the normal functions of the living organisms, given that the MNA matrix mainly serves as microenvironments for the encapsulated organisms. It is crucial to maintain the natural behaviors and metabolism of the organisms to avoid possible parasitosis or tumorigenesis. Furthermore, the new materials adopted are expected to be fine-tuned to actively program the behaviors of the integrated creatures, and therefore providing more flexible strategies. Considering biomolecules such as small peptides, glycans, and even proteins or polysaccharides, are able to provide signals that modulate cell adhesion, differentiation and development through interactions, they can be conjugated or modified on natural/artificial materials (*e.g.*, gelatin, collagen, polyethylene glycol, poly(lactic acid) and so on) through covalent bonds and are able to direct cell behaviors 115, 116. For example, PEG-based hydrogel with bone morphogenetic protein-2 (BMP-2) was employed as cell-ingrowth matrices for improving bone formation 117. (3) In addition to the sufficient mechanical strength to insert target tissues, the long-term maintenance/adhesion of MNAs on the target surface is also required. Currently, when MNAs are needed to adhere to tissues for a long period of time, the suture is usually used, which may result in significant inconvenience and infections. Therefore, new materials with durable adhesion, will greatly improve the performance of MNAs 118, 119.

#### Expanding applicable targets

Since that MNA insertion requires extra pressure using applicators or thumb-pressing, current MNA-based systems are mainly limited to the skin and heart (during surgery). Therefore, developing new strategies based on MNA is urgently demanded. For instance, when being integrated with self-triggered spring 120 or magnetic guidance 121, MNAs can be delivered to the gastrointestinal (GI) tract, indicating an enteral delivery strategy. It is expected that after encapsulating proper bacteria, those MNAs can be used to regulate the intestinal microbes. Moreover, in combination with surgeries or interventional medicine, MNAs could be applied to more organs to treat diseases or maintain homeostasis after loading specific organisms 122. For example, after being embedded with engineered liver cells, MNAs are able to attach to the liver during tumor excision surgeries, therefore helping to repair the organ or assist its functions (*e.g.*, waste clearance). With the advances of interventional medicine techniques, specific catheters can go through natural lumens (*e.g.*, arterial/venous vessels, gastrointestinal/biliary/urinary tract, *etc.*) and enable operations such as visualization, biopsy or minimally invasive surgery 123, 124. After the proper design, MNAs are expected to be delivered to the heart, liver, kidney, stomach, intestine and so on by various catheters to realize potential minimally invasive organ treatment such as lesion repairing and tumor elimination.

#### Combining new technologies

With the development of biomedical technologies, MNA functionalization would be significantly enhanced to extend their applications. For instance, molecular imaging can be integrated with MNAs by encapsulating proper imaging agents (*e.g.*, quantum dots and indocyanine green) inside MNAs, and thus facilitate visualization of pathological lesions as well as accurate medical recordkeeping 125, 126. Loading other nanoparticles that have versatile functions such as responding to environment variation 127, carrying engineered biomolecules (*e.g.*, DNA and RNA) 128, 129 and simulating cellular behaviors 130, can also enrich the functions of MNAs to improve their ability to regulate the integrated organisms.

Many emerging smart devices can be combined with MNAs as well. Microfluidics technology, acting as the newly emerging strategy to precisely control liquid motion in a small substrate 131, 132, can provide MNAs with fluid circulation capability, and therefore the MNAs are able to manipulate organism behavior by regulating the transport of nutrition and waste. Currently, several microfluidic systems combining with MNAs have been developed for various biomedical applications such as convenient and continuous injection and extraction 133, fluid management toward combinational therapy 134, and ISF collection facilitating biomarker analysis 135. Moreover, the emerging “Organ on a chip” 136-138 and organoid technology 139, 140 enable simulation or even reconstruction of intact tissue/organ. When combining with MNA technology, the “Organ on a microneedle” may open a new window to improve organism-microneedle synergistic therapies.

Recently, the technology of wearable electronics has grown pretty fast and shown promising potential in monitoring the physical/physiological status of the human body 141-143. The microscale and functional electronic devices can be implanted inside the bodies and communicate with daily-used smartphones 144, 145. Various researches have demonstrated the integration of MNAs with those smart devices. And the applications mainly focus on the fields of real-time health monitoring and controllable transdermal drug delivery 146. Similarly, MNAs would be functionalized to monitor the real-time performance of the encapsulated living organisms, ensuring the viability and status of the species. Furthermore, after assembly with microantennas to receive electric/photonic signals, and actuators (*e.g.*, circuits or chemical reactors) to exert functions, the organism behaviors are expected to be finely tuned, indicating a highly regulatory fashion. When integrating with these techniques, the biggest advantage that MNAs can offer is the fast, convenient, and low-cost biomedical implementation without the assistance from professionals, which greatly benefits point-of-care diagnosis and therapies in a personal manner beyond hospitals.

Overall, microneedle arrays integrated with living organisms have exhibited great potential in the fields of point-of-care drug administration and personalized therapies in specific diseases. Those MNAs have proven to play a significant role in vaccination and cell therapy, especially for diseases related to immune disorders, infections, tumors, tissue damage, and so on, which are difficult to treat by traditional therapies (*e.g.*, drugs or surgeries). With further development of biological, materials and engineering technologies, it is believed that the combination of living organisms with smart microneedle patches would hold great promise in the near future due to the advantages of both natural and artificial species.

## Figures and Tables

**Figure 8:**
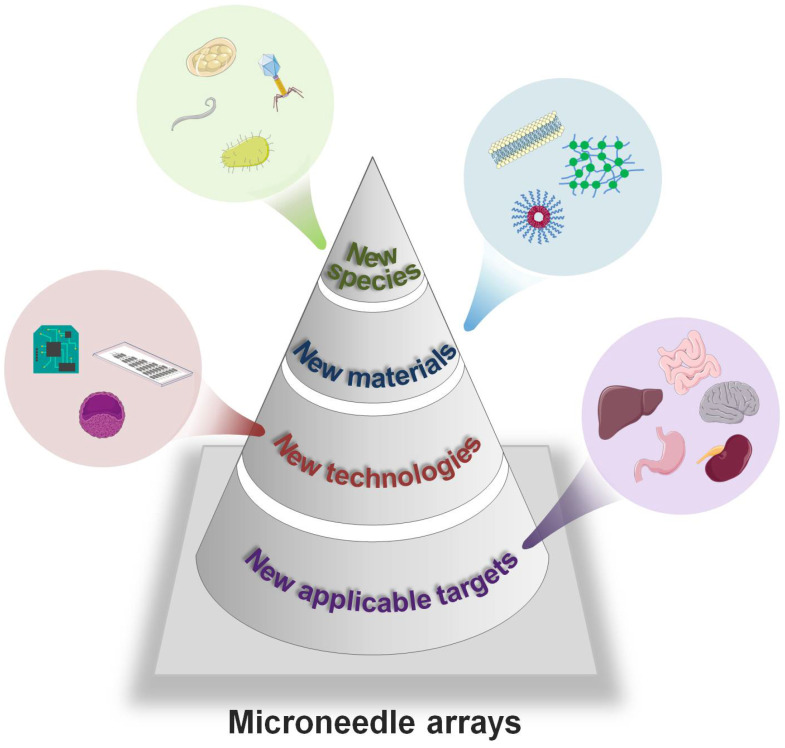
** Perspective of living organism-integrated MNAs.** The MNAs could integrate new creature species, adopt new materials, expand applicable targets and combine new technologies to further benefit human healthcare in a convenient, low-cost and point-of-care manner.

**Table 1:** MNAs integrated with viruses

Species	Microneedle arrays			Test subjects	Applications	References
	Type (T)/Materials (M)	Height (H)/Diameter (D)	Array			
Recombinant MVA	T: Bulk M: Silicon	H: 100/125/200/300 μm D: -	4 × 4 / 5 × 5 / 6 × 6 / 9 × 9 / 10 × 10 / 20 × 20	Mice	Optimizing MNA design for vaccine delivery	47
Recombinant MVA/Recombinant AdHu5/Influenza X31	T: Coated Core: Silicon Coating: Trehalose/maltodextrin/CMC/Tween 80	H: 200/300 μm D: 100/150 μm	4 × 4 / 6 × 6	Mice	Developing spray-coating method for MNA-based vaccination	48
Recombinant AdHu5	T: Dissolving M: Na-CMC/sucrose/lactose	H: 1500 μm D: 670 μm	44 needles in 1 cm^2^ substrate	Mice	Preserving bioactivity of vaccines in MNAs and showing the immune response pathways	49, 50
Recombinant MVA/Recombinant ChAd63	T: Coated Core: Silicon Coating: MC/PS20/trehalose/sucrose	H: 110 μm D: 22 μm	58 × 58	Mice	Evaluating long-term thermostability and immune response of dry-coated vaccine on MNAs	51
Recombinant MVA.ME-TRAP/Recombinant ChAd63.ME-TRAP	T: Bulk M: Silicon	H: 100/125/200/280/300 μm D: -	4 × 4 / 6 × 6 / 9 × 9 / 10 × 10 / 20 × 20	Mice	Malaria vaccine delivery by bulk MNAs	52
Recombinant AdHu5	T: Dissolving M: Trehalose	H: 500 μm D: 300 μm	5 × 5	Mice	Malaria vaccine delivery by dissolving MNAs	53
Recombinant AdHu5	T: Dissolving M: Na-CMC/sucrose/lactose	H: 1500 μm D: 670 μm	44 needles in 1 cm^2^ substrate	Mice	Skin HIV vaccine delivery inducing mobilization of long lived, poly-functional CD8^+^ T cells	54
Adeno-associated-virus 9	T: Insoluble M: PVA	H: 850 μm D: 334 μm	44 needles in a substrate (Φ6 mm)	SD rat	Gene delivery for treating ischemic myocardial disease	55
Live-attenuated measles strain Edmonston-Zagreb	T: Coated Core: Stainless steel Coating: Trehalose/CMC/Lutrol F68	H: 750 μm D: -	5 needles in a row	Cotton rats	Measles vaccination through MNAs	56
Live-attenuated measles strain Edmonston-Zagreb	T: Dissolving M: Sucrose/threonine/CMC/PVA/PMMA	H: 600 μm D: 300 μm	10 × 10	Rhesus macaques	Measles vaccination through MNAs	57
Live-attenuated Edmonston-Zagreb measles strain and RA-27 rubella strain	T: Dissolving M: Sucrose/threonine/CMC/PVA	H: 700 μm D: -	10 × 10	Rhesus macaques	Delivering MR vaccine and accessing the immunogenicity	58, 59
Live-attenuated mumps virus/Live-attenuated varicella virus	T: Bulk M: Poly-glycolic acid	H: 700 μm D: 200 μm	6 needles in a row	SD rat	Enhancing vaccine delivery and immune response	60
Live-attenuated dengue virus	T: Coated Core: Poly-L-lactide Coating: CMC/trehalose	H: 750 μm D: 250 μm	77 needles (5 × 9 + 4 × 8)	Mice	Cold chain-independent dengue vaccine storage and delivery	61
Live vaccinia virus	T: Coated Core: PLA Coating: PVA/trehalose	H: 800 μm D: 370 μm	97 needles in a substrate (Φ1 cm)	Mice	Evaluating the storage and inoculation of smallpox vaccine using MNAs	62
Cowpea mosaic virus	T: Dissolving M: PVP/Mg nanoparticles	H: 850 μm D: 400 μm	15 × 15	Mice	Melanoma treatment	63

MVA: modified vaccinia virus Ankara; AdHu5: human adenovirus serotype 5; CMC: carboxymethyl cellulose; ChAd63: Simian adenovirus Chimpanzee serotype 63; MC: methyl cellulose; PS20: polysorbate 20; ME: Multiple epitope; TRAP: thrombospondin-related-adhesive-protein; SD rat: Sprague-Dawley rat; HIV: human immunodeficiency virus; MR: measles and rubella; PVP: polyvinyl pyrrolidone; PMMA: polymethyl methacrylate.

**Table 2:** MNAs integrated with living bacteria

Species	Microneedle arrays			Test subjects	Applications	References
	Type (T)/Materials (M)	Height (H)/Diameter (D)	Array			
*C. albicans*/*P. aeruginosa*/*S. epidermidis*	T: Bulk M: Silicon	H: 280 μm D: 250 μm	6 × 7	Excised porcine skin	Evaluating microbial penetration	77
*E. coli* DH5α	T: Bulk M: Metal	H: 200 / 500 / 1000 μm D: 80 μm	7 needles per row (roller-like)	Hair-trimmed mouse skin	Evaluating potential risk of microbial infections	78
*F. novicida*	T: Bulk M: PLLA	H: 650 μm D: 250 μm	7 × 11	Mice	Inducing intradermal infections	79
Live-attenuated BCG *bacillus*	T: Dissolving M: HA	H: 200 μm D: 100 μm	6 × 9	Mice	Vaccine for tuberculosis	80
*Lactobacillus*	T: Dissolving M: SCMC	H: 600 μm D: 250 μm	9 × 9	Mice	Transdermal delivery of probiotics into local skin	81
*B. subtilis*	T: Insoluble M: PEGDA/PVA/HMPP	H: 500 μm D: 230 μm	20 × 20	Mice	Fungal infection treatment	82

PLLA: Poly(L-lactide acid); BCG *bacillus*: Bacille Calmette-Guerin* bacillus*; HA: hyaluronate; SCMC: Sodium carboxymethyl cellulose; PEGDA: poly(ethylene glycol) diacrylate; PVA: polyvinyl alcohol; HMPP: 2-hydroxy-2-methylpropiophenone.

**Table 3:** MNAs integrated with living mammal cells

Species	Microneedle arrays			Test subjects	Applications	References
	Type (T)/Materials (M)	Height (H)/Diameter (D)	Array			
HaCaT Cells/Human follicle dermal papilla cells	T: Bulk M: PMMA	H: 1000 μm D: 200 μm	6 × 6	Collagen hydrogel	Cell delivery evaluation	85
Melanocyte/Keratinocyte/Epidermal cells	T: Bulk M: Silicon	H: 400/500/600/700 μm D: 200 μm	5 × 5	Excised human breast skin	Delivering functional cells for skin healing	86
Mardin-Darby canine kidney cells	T: Bulk M: SiO_2_/Si_3_N_4_	H: 100/300/500 μm D: 30-50 μm	-	Rat liver tissue	Cell transplantation	87
B16-F10-murine-melanoma/HEK-293T cells	T:Dissolving M: HA/PVP/maltose	H: 650 μm D: 300 μm	10 × 10	Mice	*In vivo* tumor grafting	88
MSCs	T: Insoluble M: PLGA/GelMA	H: 700 μm H/D ratio = 1.5	8 × 8	Mice	Wound repairing	89
RFP-HeLa/HaCaT/NDFs/MSCs/melanocytes/T cells/bone marrow-derived DCs/LPS-treated DCs	T: Dissolving M: PBS/DMSO/sucrose	H: 900 μm D: 350 μm	10 × 10	Mice	Transdermal delivery of therapeutic living cell	90
Pancreatic β-cells	T: Insoluble M: HA/alginate	H: 800 μm D: 400 μm	20 × 20	Mice	Type-1 diabetes treatment	91
CSCs	T: Insoluble M: PVA/fibrin gel	H: 600 μm D: 300 μm	20 × 20	Rat/Porcine	Heart regeneration after acute myocardial infarction	92
iPSC-derived cardiomyocytes	T: Insoluble M: GelMA/CNT	-	-	Mice	Treating acute myocardial infarction	93
CAR T cells	T: Solid M: PLGA	H: 1500 μm D: 250 μm	15 × 15	Mice	Seeding of CAR T cells for augmenting anticancer efficacy	94

PMMA: polymethyl methacrylate; HA: hyaluronate; PVP: polyvinyl pyrrolidone; MSC: Mesenchymal stem cells; PLGA: poly(lactic-co-glycolic acid); GelMA: gelatin-methacryloyl; RFP: red fluorescent protein; NDF: Normal dermal fibroblast; DC: Dendritic cell; PBS: Phosphate buffer saline; DMSO: dimethylsulfoxide; CSC: cardiac stromal cell; PVA: polyvinyl alcohol; iPSC: induced pluripotent cell; CNT: carbon nanotube; CAR: chimeric antigen receptor.
